# Molecular Dynamics Simulation and Experimental Study on the Mechanical Properties of Functionalized Graphene-Enhanced PEEK/PTFE

**DOI:** 10.3390/polym18010125

**Published:** 2025-12-31

**Authors:** Yan Wang, Jingjing Chen, Henan Tang, Bin Yang, Shijie Wang, Ning Wang

**Affiliations:** School of Mechanical Engineering, Shenyang University of Technology, Shenyang 110870, China

**Keywords:** polytetrafluoroethylene, polyether ether ketone, functionalized graphene, molecular dynamics, mechanical properties

## Abstract

The reinforcement mechanism of functionalized graphene nanosheets (GNS) on the mechanical properties of polyetheretherketone (PEEK)/polytetrafluoroethylene (PTFE) composites was investigated. Composite specimens were fabricated using PGNS, as well as GNS grafted with hydroxyl, carboxyl (-COOH) and amino functional groups, and mechanical characterizations were conducted on the prepared specimens. The results demonstrated that carboxyl-functionalized GNS (COOH-GNS) exhibited the most remarkable reinforcing effect on PEEK/PTFE composites, with its elastic modulus, tensile strength, yield strength and compressive modulus increased by 47.09%, 31.1%, 45.16% and 20.91%, respectively, compared with PGNS-reinforced composites. Combined with experimental measurements and molecular dynamics simulations, the reinforcement mechanism of this composite system was elucidated. The functional groups on the surface of GNS can induce interfacial interactions with the PEEK/PTFE matrix, by which the mobility of polymer molecular chains is restricted, the deformation and slippage of molecular chains are suppressed, and the interfacial bonding between GNS and the polymer matrix is simultaneously strengthened. The enhancement of interfacial binding energy, the reduction in free volume in the composite system, and the restriction of polymer molecular chain mobility were identified as the critical atomic-scale mechanisms responsible for the improvement of the macroscopic mechanical properties of the composites.

## 1. Introduction

Polytetrafluoroethylene (PTFE) is extensively utilized in engineering as a type of sealing and lubricating material owing to its outstanding high- and low-temperature resistance, chemical stability, and low friction coefficient [1,2]. However, its inferior mechanical properties, including low hardness and poor wear resistance, attributed to the unique molecular structure, significantly restrict practical applications under heavy-load and high-speed friction conditions [3]. Extensive investigations have been conducted to enhance the mechanical properties and hardness of PTFE composites. Various fibers, metal particles, and special engineering plastics are commonly used reinforcing fillers for PTFE [4,5,6].

Polyetheretherketone (PEEK) is a semicrystalline, thermoplastic specialty engineering plastic exhibiting excellent corrosion resistance, fatigue resistance, lightweight characteristics, and cost-effectiveness. It is widely utilized in severe mechanical friction environments and aerospace applications. The superior properties of PEEK make it a common matrix material for PTFE filled composites, with optimal wear resistance and mechanical performance achieved at 10 wt% PEEK content [7,8].

These filler materials are generally constrained by factors such as size, interfacial adhesion, and compatibility, resulting in unsatisfactory reinforcement effects [9,10]. Consequently, the incorporation of nano reinforcement materials into polymer composites has emerged as an important topic in recent years. New nanomaterials include fullerenes, carbon nanotubes, graphene nanosheets (GNS), and their derivatives. GNS are composed of single-layer sp^2^-hybridized carbon atoms arranged in a hexagonal honeycomb lattice structure, exhibiting large specific surface area, high elastic modulus, and exceptional electrical and thermal conductivity [11]. GNS have been utilized to modify various polymer materials. Sepetcioglu et al. [12] found that GNS generates frictional sliding within the matrix and synergistically interact with fiber interfaces, resulting in a 31% increase in fracture toughness. Srivastava et al. [13] confirmed that the incorporation of 1.0 wt% graphene GNS enabled efficient stress transfer and significantly improved the tensile and flexural strength of the composites. Li et al. [14] investigated the mechanical properties of GNS-enhanced polymer matrix composites, revealing that GNS incorporation substantially enhanced both mechanical and tribological performance of the polymer composites. The studies indicate that incorporating a small amount of GNS into polymer materials can not only significantly enhance their mechanical properties but also retain the inherent properties of the polymers themselves. However, there are strong van der Waals (vdW) forces between the layers of pure GNS, which lead to severe aggregation and poor dispersion in the polymer matrix—a problem that greatly limits their lubricating and reinforcing effects [15,16]. Therefore, the optimal incorporation of GNS into polymer matrices to simultaneously preserve the intrinsic performance of polymers and significantly enhance their mechanical properties has become the focus of our research.

Chemical modification introducing specific functional groups onto GNS surfaces can effectively suppress aggregation, enhance interfacial bonding with polymer matrices, and substantially improve the mechanical properties of polymer nanocomposites [17,18,19,20,21]. Amit et al. [22] experimentally confirmed that functionalized GNS significantly enhanced the interlaminar shear strength and tensile strength of carbon fiber/epoxy composites. Li et al. [23] revealed that functionalized GNS modified the thermomechanical and mechanical properties of low-density polyethylene. Wu et al. [24] showed that oxygen-rich functional groups on GNS enhance interfacial interactions with PHA molecular chains. While these experimental studies have convincingly demonstrated the macroscopic enhancement effects of functionalized GNS on various polymer composites, the underlying atomic-scale mechanisms governing the interfacial interactions and mechanical behavior in specific systems such as PEEK/PTFE remain incompletely elucidated. To bridge this knowledge gap, molecular dynamics (MD) simulations emerge as a powerful tool to quantitatively probe the nanoscale reinforcement mechanisms.

Recent extensive research efforts have demonstrated the successful application of MD simulations in elucidating the mechanical behavior of polymer nanocomposites. Juma et al. [25] systematically investigated the reinforcement mechanisms of GNS and functionalized GNS as nanofillers in polypropylene, polyethylene, and polyvinyl alcohol composites through MD simulations. Samanta et al. [26] employed MD simulations to elucidate the effects of GNS nanofillers on the mechanical and thermophysical properties of polyvinyl alcohol composites. Heydari et al. [27] systematically investigated the influence of oxidized GNS on the mechanical performance of polyurethane/polycaprolactone nanocomposites through MD simulations. Xu et al. [28] employed MD simulations to quantitatively analyze critical parameters. This approach established a quantitative relationship between functional group modifications and macroscopic mechanical properties at the atomic scale. MD simulations can be employed to analyze the effects of various modification methods on material performance. Hildebrand et al. [29] demonstrated that interactive visualization and web-based sharing of MD simulation data improve the interpretability, reliability, and reusability of simulation studies. Qiao et al. [30] combined reciprocating dry-sliding experiments with MD simulations to elucidate the wear mechanisms of a Fe_2.5_Ni_2.5_CrAl multi-principal element alloy. Zhang et al. [31] used MD simulations to study the mechanical properties and failure mechanisms of multilayer graphene oxide nanosheets; their simulated parameters showed close agreement with experimental measurements. This discovery provides a crucial perspective for the current investigation.

Building on the successful application of MD simulations in polymer nanocomposite research and related findings, this work further investigates the modification of the PEEK/PTFE composite system with functionalized GNS. According to the findings of Xie et al. [32], the incorporation of GNS into PTFE can effectively enhance its mechanical performance: at a GNS loading of 2 wt%, the strength and elongation at break of the material are increased by 21.5% and 28.6%, respectively. Building on these results, this study introduces 2 wt% functionalized GNS into the 10% PEEK/PTFE composite system to systematically investigate the regulatory effects of functionalized GNS on the mechanical properties of this hybrid material. Specifically, the respective mass fractions of GNS, PEEK, and PTFE in the composite are 2 wt%, 9.8%, and 88.2%.

In this study, different types of hydrophilic functional groups were selected to modify GNS for comparative analysis of their regulatory effects on the composite system. Specifically, pristine GNS (PGNS) and GNS modified with carboxyl (-COOH), amino (-NH_2_), and hydroxyl (-OH) groups were utilized to fabricate four composite formulations, followed by relevant mechanical property tests. Through mechanical characterization of these four composite groups combined with MD simulation analysis, this study elucidates the microscopic mechanisms underlying the macroscopic mechanical experimental results. By analyzing the surface morphology of functionalized GNS as well as key atomic-scale parameters including interfacial binding energy, free volume (FV), mean squared displacement (MSD), and radial distribution function (RDF), this work clarifies the regulatory effects of functional group types on the dispersion behavior and interfacial interaction mechanisms of GNS within the PEEK/PTFE matrix, and identifies the enhancement effects and intrinsic mechanisms of different functionalization modification strategies on the mechanical properties of the composites.

## 2. Experiment and Modeling

### 2.1. Experiment Materials

PTFE was procured from the Japan Daikin Fluorine Chemical Co., Ltd., (Osaka, Japan) with an average particle size of 40 μm and a density of 2.14 to 2.20 g/cm^3^. PEEK was procured from the Jilin Zhongyan Polymer Materials Co., Ltd. (Changchun, China), with an average particle size of 24 μm and a density of 1.4 g/cm^3^. The functionalized GNS were procured from the Shenzhen Guoheng Qihang Technology Co., Ltd., (Shenzhen, China) and they featured a multilayer structure with an average nanoplatelet diameter of 25 μm.

### 2.2. Sample Preparation

PTFE and PEEK powders were baked at 150 °C for 3 h and sealed. The composites were formulated with a constant matrix composition of 88.2 wt% PTFE and 9.8 wt% PEEK. Four different types of GNS were incorporated at a fixed loading of 2 wt%: PGNS, HO-GNS, COOH-GNS, and NH_2_-GNS. The composite containing PGNS is labeled as PGNS/PEEK/PTFE, while the composite containing functionalized GNS was labeled as HO-GNS/PEEK/PTFE, COOH-GNS/PEEK/PTFE, and NH_2_-GNS/PEEK/PTFE, respectively. They were then mixed in a high-speed blender at 1200 rpm for 60 s per cycle, and this process was repeated 10 to 15 times. After mixing thoroughly, the samples were cooled to an ambient temperature and sealed. Subsequently, the blended materials were cold pressed in a hydraulic press at 30 MPa for 30 min. They were left undisturbed for more than 12 h to release internal stress. Subsequently, the compact materials (referred to as compact) were placed in an oven and heated from room temperature to 360 °C at a rate of 50 °C/h for 3 to 4 h. Finally, the compacted materials gradually cooled to room temperature (25 °C) and then machined to obtain the desired samples.

### 2.3. Experimental Methods and Characterization

Tensile and compression tests were performed on an AG-X series universal testing machine (Shimadzu Corporation, Kyoto, Japan). The tensile tests were conducted in accordance with the Chinese standard GB/T 1040.2-2022 [33], “Plastics—Determination of tensile properties—Part 2: Test conditions for molding and extrusion plastics” The compression tests followed the Chinese standard GB/T 1041-2008 [34], “Plastics—Determination of compressive properties”. For the tensile tests, the specimens had a diameter of 6 mm (±0.4 mm) in the reduced section of the gauge length and a gauge length of 33 mm (±2 mm). The overall length of the tensile specimens was 80 mm. The specimens for compression tests were prepared with dimensions of 10 mm × 10 mm × 4 mm. The crosshead speed was set to 50 mm/min for the tensile tests and 2 mm/min for the compression tests. All mechanical testing data are presented as the average values of each group to enhance the reliability of the results. The specimens were sputter-coated with a gold layer. Scanning electron microscopy (SEM) and energy-dispersive X-ray spectroscopy (EDS) analyses were subsequently performed using a Zeiss Sigma 300 scanning electron microscope (Carl Zeiss AG, Oberkochen, Germany). The mechanical properties of the composite materials were analyzed from tensile resistance and compressive resistance perspectives. The elastic modulus, tensile strength, yield strength under tensile loading, and compressive modulus during compression were systematically evaluated to elucidate the composite’s mechanical performance by comparing the composites incorporated with differently functionalized GNS/PEEK/PTFE specimens.

### 2.4. Simulation Method

#### 2.4.1. Modeling and Optimization

MD simulations were performed using Material Studio 2018 software on a 16-core AMD Ryzen 7 4800H processor (Advanced Micro Devices, Inc., Santa Clara, CA, USA) with Radeon Graphics. Amorphous molecular models were constructed for GNS, HO-GNS, COOH-GNS, and NH_2_-GNS-filled PEEK/PTFE composites.

**Figure 1:**
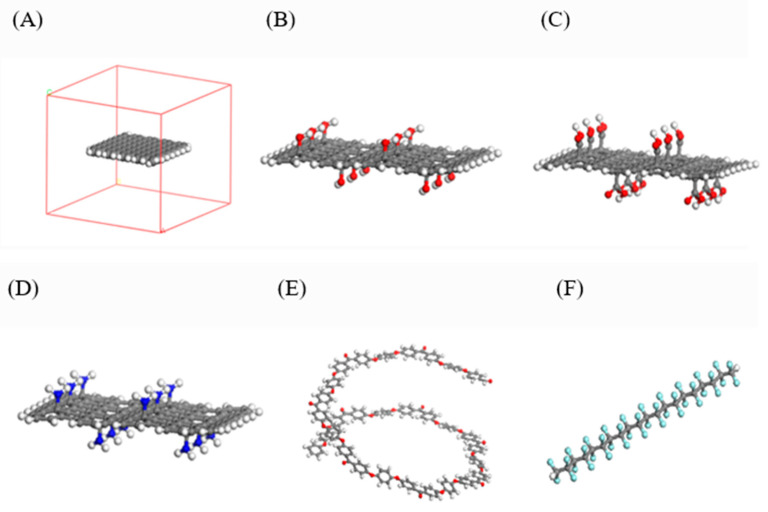
Configurations of (**A**) periodic lattice, (**B**) HO-GNS, (**C**) COOH-GNS, (**D**) NH_2_-GNS, (**E**) PEEK, and (**F**) PTFE models.

First, one piece of single-layer GNS with dimensions of 1.98 × 1.66 nm^2^ was constructed and placed at the center of a periodic lattice with a size of 3.3 × 3.3 × 3 nm^3^, and its configuration is illustrated in Figure 1A. For the HO-GNS, 12 hydroxyl functional groups were uniformly grafted onto both the top and bottom surfaces of the other GNS, with the corresponding structure presented in Figure 1B. Using the same grafting strategy, COOH-GNS and NH_2_-GNS were fabricated separately, and their configurations are shown in Figure 1C,D, respectively. To eliminate the interference of unsaturated boundary conditions on the simulation results, the edges of all GNS were passivated via hydrogen atom grafting [35]. Subsequently, the three types of functionalized GNS were individually placed at the center of periodic lattices with the same specifications as described above. Meanwhile, PEEK and PTFE molecular chains with a degree of polymerization of 10 were constructed, and their molecular chain structures are displayed in Figure 1E,F. Finally, the Monte Carlo “random number [36]” algorithm combined with the Amorphous Cell module was employed to randomly pack the PEEK and PTFE molecular chains into the periodic lattices containing various GNS at a preset density of 2.1 g/cm^3^. The resulting lattice system consists of 1 PEEK molecular chain, 26 PTFE molecular chains, and 1 piece of GNS, with the mass fractions of GNS, PEEK, and PTFE consistent with the experimental formulation.

**Figure 2:**
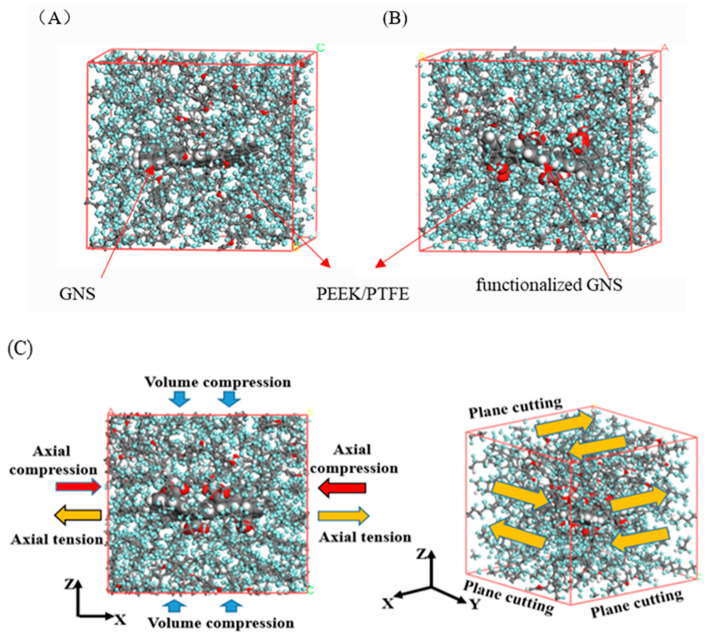
Equilibrated configurations of GNS/PEEK/PTFE composites and constant strain method. (**A**) GNS/PEEK/PTFE equilibrium geometries. (**B**) Functionalized GNS/PEEK/PTFE equilibrium geometries. (**C**) Schematic diagram of the constant strain method.

The amorphous molecular models of the composites were subjected to geometric optimization and MD equilibration to obtain global and local minimum energy configurations. Geometric optimization was performed using the steepest descent and conjugate gradient methods with an energy convergence criterion of 10^−5^ kJ/mol. Two-stage molecular dynamics equilibration was conducted to eliminate internal stress within the molecular systems. The first equilibration was performed under the NVT ensemble at 298 K using the Andersen thermostat, with a simulation step size of 1 fs and a total duration of 0.5 ns. Subsequently, a 1 ns isothermal-isobaric (NPT) ensemble molecular dynamics simulation was performed under the conditions of 298 K and 101 kPa. The system was deemed to have reached a stable equilibrium state when the fluctuation ranges of temperature, pressure and potential energy were within 5% after equilibration. All calculations employed the COMPASS [37,38] force field, with vdW interactions calculated using the Atom-based method and electrostatic interactions computed via the Ewald method, adopting a cutoff radius of 1.25 nm. The fully equilibrated system configurations are illustrated in Figure 2A,B.

#### 2.4.2. Mechanical Property Characterization

The lowest-energy configuration obtained from the NPT dynamics equilibration simulation described in the previous section was selected. Taking the last frame of this configuration as the initial state, a further 200 ps NVT molecular dynamics simulation was performed, with all simulation parameters kept consistent with those used in the initial NVT dynamics stage. Subsequently, the constant strain method was adopted to calculate the mechanical properties of the stable configurations extracted from the last 40 ps of the trajectory file. The detailed procedure is illustrated in Figure 2C. The composite system was subjected to uniaxial tensile loading across six deformation modes (*x*, *y*, *z*, *xy*, *xz*, *yz*) through four incremental strain cycles with *ε* = (−0.003, −0.001, 0.001, 0.001, 0.003), where the maximum strain amplitude reached 0.003. The stress components of the composite material in each direction are calculated based on the definition of virial stress. The *E*_i_ is derived using the relationships: *E*_i_ = *σ*_1_/*ε*_1_, *σ*_1_ = *C*_ij_/*ε*_j_ and *ε*_1_ = *S*_ij_*σ*_j_, where *σ*_1_ represents stress, *ε*_1_ denotes strain, *C*_ij_ is the stiffness matrix, and *S*_ij_ is the compliance matrix. The macroscopic bulk modulus and shear modulus of the composite material are derived from the stiffness matrix (*C*_ij_) obtained through MD simulations. The overall equivalent modulus of the composite system is then estimated using the Voigt–Reuss–Hill (VRH) averaging scheme. The bulk modulus (B) and shear modulus (G) of the composite were calculated using Equations (1)–(4). Based on the VRH, the actual bulk modulus (*B*_H_) and shear modulus (*G*_H_) of the composite system were derived from Equations (5) and (6), with the results presented as follows.

(1) $$B_{V}=\frac{1}{9}\left(\sum_{i=1}^{3}C_{\mathrm{ii}}+2\sum_{\begin{array}{l} i=1\\ i<j \end{array}}^{3}C_{\mathrm{ij}}\right)$$

where *C*_ij_ denotes the stiffness matrix, where the subscripts i and j range from 1 to 6, representing the six independent stress and strain components (specifically, 1, 2, 3 correspond to normal stresses and strains along the *x*, *y*, *z* directions, while 4, 5, 6 correspond to shear stresses and strains).

(2) $$G_{V}=\frac{1}{15}\left[\left(\sum_{i=1}^{3}C_{\mathrm{ii}}-\sum_{\begin{array}{l} i,j=1\\ i<j \end{array}}^{3}C_{\mathrm{ij}}\right)+3\sum_{i=4}^{6}C_{\mathrm{ii}}\right]$$

where *S*_ij_ is the compliance matrix, which is the inverse of the stiffness matrix and describes the ease with which a material deforms under stress.

(3) $$B_{R}={\left(\sum_{i=1}^{3}S_{\mathrm{ii}}+2\sum_{\begin{array}{l} i,j=1\\ i<j \end{array}}^{3}S_{\mathrm{ij}}\right)}^{-1}$$

where *B*_R_ is the Reuss bulk modulus. It is calculated based on the assumption of stress uniformity and typically represents the lower bound of the equivalent modulus.

(4) $$G_{R}=\frac{15}{4\left(\sum_{i=1}^{3}S_{\mathrm{ii}}-\sum_{\begin{array}{l} i,j=1\\ i<j \end{array}}^{3}S_{\mathrm{ij}}\right)+3\sum_{i=4}^{6}S_{\mathrm{ii}}}$$

where *G*_R_ is the Reuss shear modulus. It is calculated based on the assumption of stress uniformity and represents the lower bound of the equivalent shear modulus.

(5) $$B_{H}=\frac{1}{2}\left(B_{V}+B_{R}\right)$$

where the *B*_H_ represents the equivalent bulk modulus, defined as the arithmetic average of the Voigt and Reuss bounds according to the VRH.

(6) $$G_{H}=\frac{1}{2}\left(G_{V}+G_{R}\right)$$

where the *G*_H_ represents the effective shear modulus, defined as the arithmetic average of the Voigt and Reuss bounds according to the VRH.

## 3. Results and Discussion

### 3.1. Mechanical Properties

**Figure 3:**
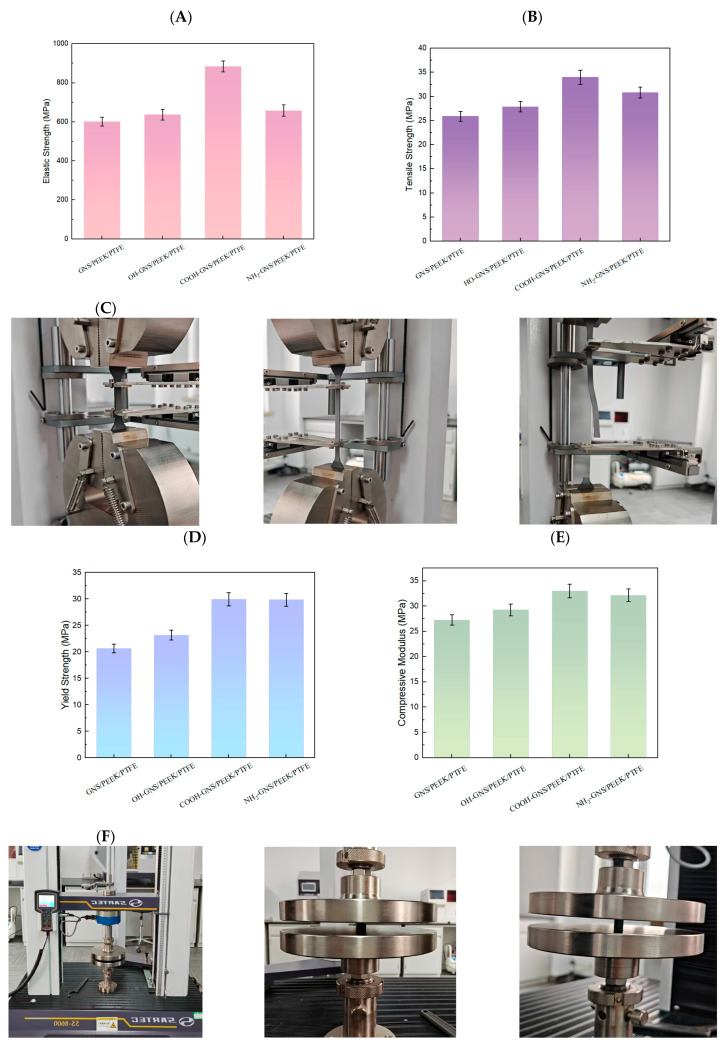
Tensile test results of the GNS/PEEK/PTFE composite with error bars indicating the 95% confidence interval: (**A**) Elastic modulus, (**B**) Tensile strength, (**C**) Tensile test procedure, (**D**) Yield strength, (**E**) Compressive modulus, (**F**) Compression test procedure.

The mechanical properties of the composites were evaluated based on the elastic modulus, tensile strength, and yield strength (obtained from tensile tests) as well as the compressive modulus (obtained from compression tests) of samples reinforced with different functionalized GNS. We adopt the 95% confidence intervals (CIs) are used to reflect the statistical significance of these mechanical property data [39,40]. The results for each composite are derived from three parallel experiments and are summarized in Figure 3.

The composites containing functionalized GNS exhibited improvements in elastic modulus, tensile strength, yield strength, and compressive modulus compared to those reinforced with PGNS as shown in Figure 3. The HO-GNS, COOH-GNS, and NH_2_-GNS/PEEK/PTFE composites exhibited elastic modulus values of 637 MPa, 884 MPa, and 658 MPa, respectively. These values correspond to enhancements of 5.99%, 47.09%, and 9.48% when compared to the composite containing PGNS (601 MPa). It can be observed from the data that COOH-GNS provides the most significant enhancement effect on the elastic modulus of the composite. The HO-GNS, COOH-GNS, and NH_2_-GNS/PEEK/PTFE composites exhibited tensile strength values of 27.91 MPa, 33.98 MPa, and 30.82 MPa, respectively. These values correspond to enhancements of 7.68%, 31.10%, and 18.90% compared to the composite containing PGNS (25.92 MPa). The data indicates that COOH-GNS exhibits superior tensile enhancement effects on the composite material. The yield strength values of HO-GNS, COOH-GNS, and NH_2_-GNS/PEEK/PTFE composites were measured as 23.19 MPa, 29.96 MPa, and 29.84 MPa, respectively. These values represent enhancements of 12.35%, 45.16%, and 44.57% compared to the composite reinforced with PGNS (20.64 MPa). This finding demonstrates that both COOH-GNS and NH_2_-GNS can effectively enhance the yield strength of the composite material. The compressive modulus values of HO-GNS, COOH-GNS, and NH_2_-GNS/PEEK/PTFE composites were determined to be 29.27 MPa, 32.96 MPa, and 32.16 MPa, respectively. These values correspond to enhancements of 7.37%, 20.91%, and 17.98% relative to the composite filled with PGNS (27.26 MPa). The data indicates that COOH-GNS provides the most significant enhancement to the compressive modulus of the composite. It can be inferred that functionalized GNS can play a role in skeletal support, improving the load-bearing capacity and shear resistance of the composite material. Among them, the COOH-GNS/PEEK/PTFE composite exhibits the optimal elastic modulus, compressive modulus, and yield strength. This may be because the introduction of -COOH functional groups better improves the dispersibility of GNS in the matrix.

### 3.2. SEM and EDS Analysis

In this study, SEM and EDS analyses were performed to thoroughly investigate the agglomeration behavior of GNS during the blending process and the interfacial bonding state of the fractured surfaces, as well as to verify the relevant hypotheses proposed in the preceding sections.

**Figure 4:**
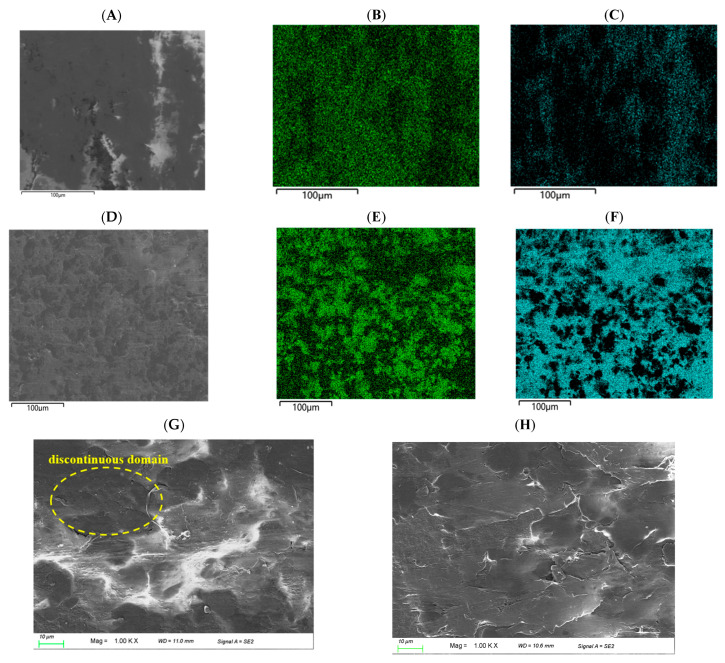
EDS characterization and SEM morphologies of PGNS/COOH-GNS reinforced PEEK/PTFE composite surfaces: (**A**) SEM morphology of GNS/PEEK/PTFE, (**B**) C element distribution of GNS/PEEK/PTFE, (**C**) F element distribution of GNS/PEEK/PTFE, (**D**) SEM morphology of COOH-GNS/PEEK/PTFE, (**E**) C element distribution of COOH-GNS/PEEK/PTFE, (**F**) F element distribution of COOH-GNS/PEEK/PTFE, (**G**) SEM morphology of GNS/PEEK/PTFE fracture surfaces, (**H**) SEM morphology of COOH-GNS/PEEK/PTFE fracture surfaces.

EDS was employed to gain deeper insight into whether GNS agglomerate during the blending process and verify the speculation from the previous section. By detecting elemental distribution via EDS, crucial evidence can be provided for determining the dispersion state of GNS in the blending system. SEM (model JSM-IT800 JEOL Ltd., Akishima, Tokyo, Japan,) coupled with energy-dispersive EDS (model Oxford Xplore 30, Oxford Instruments plc, Abingdon, Oxfordshire, UK) was employed to characterize the distribution of C and F elements across the two specimen groups. Comparative results are presented in Figure 4. Given the presence of C in all components (GNS, PEEK, and PTFE), the distribution of C does not provide a reliable indicator for evaluating material dispersion. A comparative inverse analysis was performed focusing only on the distribution of F, which is the most abundant element next to C in this material. Observation of the F distribution across the two image sets reveals discontinuous dispersion of F in Figure 4A, accompanied by extensive porous structures. This suggests that the pore regions are occupied by C from GNS or by C, H, and O from PEEK, indicating probable aggregation of graphene at these porous locations. The distribution of F in Figure 4B is relatively uniform and continuous. C from functionalized GNS, as well as C, H, and O from PEEK, are homogeneously distributed within smaller cavities, indicating that functionalized GNS disperses more uniformly in the PEEK/PTFE matrix compared to PGNS.

The surface morphologies of the tensile fracture interfaces of two groups of specimens were characterized, as presented in Figure 4G,H. As observed from Figure 4G, although no severe agglomeration is detected on the fracture interface, slight discontinuous domains are present in the texture of local regions, indicating that the dispersion of PGNS in the matrix is relatively poor. In contrast, the fracture interface in Figure 4H exhibits continuous and uniform rough lamellar textures with no large-scale agglomerates observed, demonstrating that functionalized GNS can be dispersed more uniformly in the PEEK/PTFE matrix. In addition, the fracture interface in Figure 4G exhibits a slightly higher density of pores and gaps than that in Figure 4H. This observation indicates that the interfacial interaction between PGNS and the matrix is relatively weak, which gives rise to localized debonding at the GNS-matrix interface, and indirectly reflects the inadequate dispersion uniformity of PGNS within the matrix. As depicted in Figure 4H, the fracture surface of the COOH-GNS/PEEK/PTFE composite is dominated by intrinsic layered peeling of the matrix, with no distinct debonding pores induced by graphene agglomerates observed. This phenomenon demonstrates that the well-dispersed carboxylated GNS constructs a continuous micro-skeleton throughout the PEEK/PTFE matrix. Under external loading, this micro-skeleton can effectively bridge the matrix phase, bear a portion of the applied stress, and consequently impart enhanced mechanical properties to the composite.

### 3.3. Modulus Simulation Analysis

Experimental studies confirm that COOH-GNS are the most effective enhancer of the mechanical properties of polyether ether ketone PEEK/PTFE composites. To elucidate the underlying mechanism, this study employs MD simulations to analyze the atomic scale reinforcement provided by COOH-GNS. Three independent structural models were constructed for each composite system, and parallel simulations were carried out using the COMPASS force field under consistent relaxation and computational parameters to ensure statistical reliability. We adopt the 95% CIs to reflect the statistical significance of the results [39,40]. The results reveal the reinforcement mechanism of functionalized GNS from the perspectives of atomic interactions, interfacial bonding, and polymer chain dynamics, thereby offering broad theoretical support for the design and optimization of PTFE-based composites.

**Figure 5:**
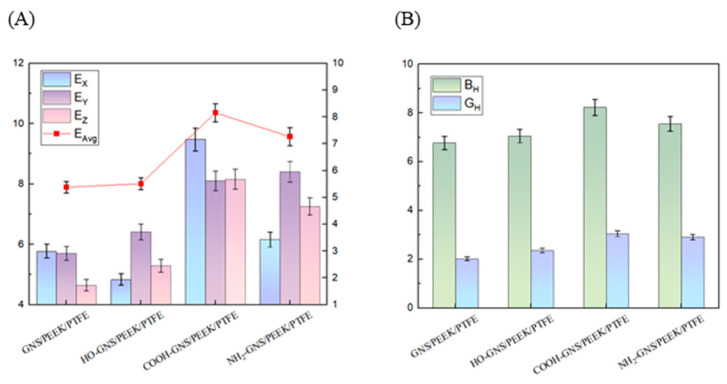
Mechanical properties of GNS/10%PEEK/PTFE composites with error bars indicating the 95% confidence interval (GPa): (**A**)Young’s modulus; (**B**) Bulk modulus and Shear modulus.

*E*_X_, *E*_Y_ and *E*_Z_ denote in the x, y and z directions, respectively; *E*_Avg_ is the average *E*_i_; *B*_R_ and *B*_V_ are the *R*_euss_ bulk modulus and *V*_oigt_ bulk modulus, respectively; *G*_R_ and *G*_V_ are the *R*_euss_ shear modulus and *V*_oigt_ shear modulus, respectively. Functionalized GNS significantly enhanced the Young’s modulus, shear modulus, and bulk modulus of the composite material along the *x*, *y*, and *z* directions, as evidenced by Figure 5. The PEEK/PTFE composites filled with HO-GNS, COOH-GNS, and NH_2_-GNS exhibited average *E*_Avg_ values of 5.51 GPa, 8.58 GPa, and 8.41 GPa, respectively. These results indicate corresponding enhancements of 2.41%, 59.48%, and 56.32% compared to the PGNS/PEEK/PTFE composite (5.38 GPa). The shear modulus values of PEEK/PTFE composites filled with pure GNS, HO-GNS, COOH-GNS, and NH_2_-GNS were measured as 2.02 GPa, 2.36 GPa, 3.01 GPa, and 2.87 GPa, respectively, as presented in Figure 5. These data demonstrate that functionalization treatment of GNS resulted in respective enhancements of shear modulus by 16.83%, 49.00%, and 42.08 percentage points.

The bulk modulus values of HO-GNS, COOH-GNS, and NH_2_-GNS reinforced PEEK/PTFE composites were measured as 7.06 GPa, 8.23 GPa, and 7.55 GPa, respectively, as presented in Figure 5. The bulk modulus values of the three functionalized GNS composites demonstrated respective increases of 4.28%, 21.57%, and 11.52% points compared to the pure GNS/PEEK/PTFE composite (6.77 GPa). It is evident that functionalized GNS significantly enhanced the overall mechanical properties of PEEK/PTFE composites, with COOH-GNS demonstrating the most pronounced enhancement in both mechanical performance and resistance to volumetric deformation. This trend is consistent with the experimental results of elastic modulus and compressive modulus in mechanical property parameters, and the influence mechanism of functionalized GNS on the mechanical properties of the composites will be further analyzed in the following text.

**Figure 6:**
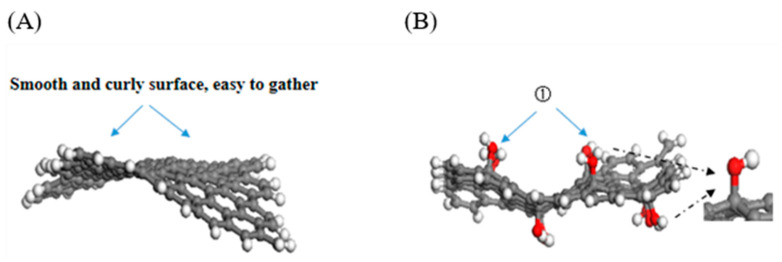

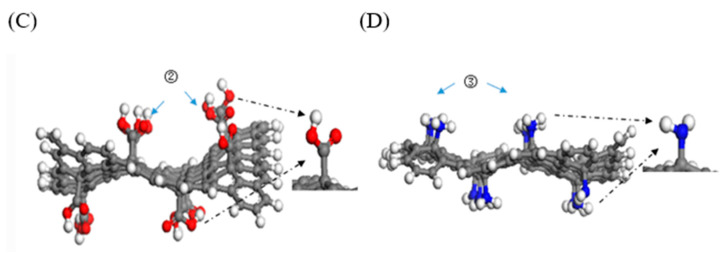
Equilibrated GNS configurations in the PEEK/PTFE composites: (**A**) PGNS, (**B**) HO-GNS, (**C**) COOH-GNS, and (**D**) NH_2_-GNS. ①②③: Rough surface, with wrinkles and less curling.

Morphological analysis of both pristine and functionalized GNS was conducted to investigate the mechanisms by which functionalized GNS influence the mechanical properties of the composite matrix, as illustrated in Figure 6. The incorporation of functionalized GNS into the PEEK/PTFE matrix resulted in a more pronounced disparity in *E*_i_ between the *x*, *y* and *z* directions, indicating enhanced anisotropy of the composite material. Observation of the equilibrated structures in Figure 6 reveals that the PGNS exhibits a relatively smooth surface and curled morphology, which contributes to its tendency for aggregation. In contrast, C atoms bonded to functional groups on modified GNS surfaces underwent a transition from sp^2^ to sp^3^ hybridization following functionalization, resulting in a slightly distorted tetrahedral configuration [41]. The grafting of multiple functional groups induced surface wrinkling through the formation of tetrahedral configurations, consequently enhancing surface roughness and in-plane stiffness [42], effectively suppressing the curling and aggregation tendencies of GNS. Therefore, functionalized GNS exhibits superior stability, enhanced dispersibility, and higher in-plane (XY-plane) stiffness in the PEEK/PTFE matrix compared with PGNS, thereby endowing the PEEK/PTFE composites with better resistance to external forces. Meanwhile, when the PEEK/PTFE composites are subjected to Z-direction (transverse) loading, the interaction between functionalized GNS and the PEEK/PTFE matrix is primarily achieved through the adsorption of functional groups. To verify the above conclusions, the interfacial binding energy between GNS and the PEEK/PTFE matrix during NVT dynamic equilibrium was calculated using Equation (7), and the relevant results are presented in Figure 6.

The revised content is strictly consistent with the experimental and simulation data reported in the manuscript, eliminating subjectively speculative descriptions and enhancing the scientific rigor and rationality of the conclusions. We have also carefully checked other parts of the manuscript to avoid similar issues.

Interfacial binding energy represents the energy required to overcome the interfacial interactions between reinforcing materials and the matrix, thus eliminating their mutual adhesive forces. To wit:

(7) $$U_{\mathrm{bind}}=-U_{\mathrm{int}\mathrm{er}}=\;\left(U_{\mathrm{total}}-U_{\mathrm{PEEK}/\mathrm{PTFE}}-U_{\mathrm{GNS}}\right)$$

where *U*_bind_ denotes the interfacial binding energy between GNS and the PEEK/PTFE matrix; *U*_inter_ represents the interfacial interaction energy; *U*_total_ is the total energy of the GNS/PEEK/PTFE composite; *U*_PEEK/PTFE_ corresponds to the energy of the PEEK/PTFE matrix; and *U*_GNS_ is the energy of GNS.

**Figure 7:**
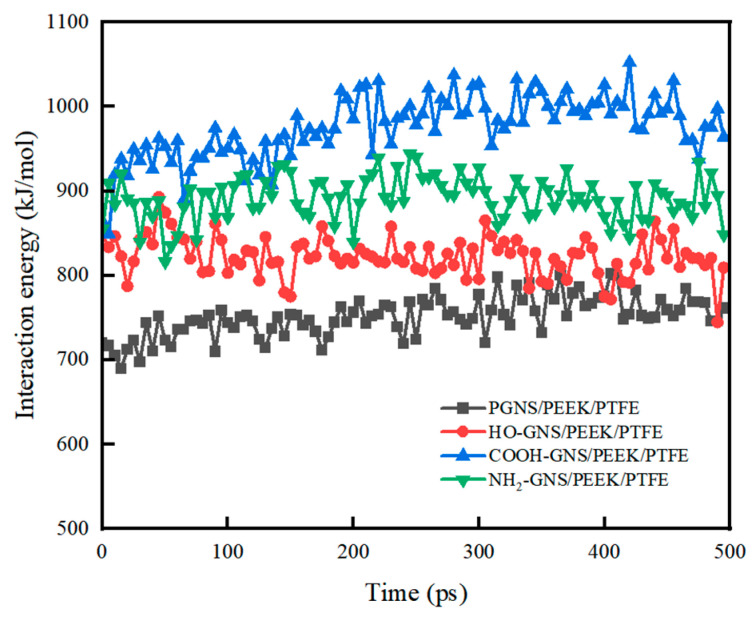
Interfacial binding energy between the GNS and the PEEK/PTFE matrix.

The average interfacial binding energies between GNS, HO-GNS, COOH-GNS, and NH_2_-GNS with the PEEK/PTFE matrix were determined to be 185.93 kcal/mol, 203.66 kcal/mol, 241.46 kcal/mol, and 220.72 kcal/mol, as illustrated in Figure 7. These results demonstrate that compared to PGNS, the functionalized GNS composites exhibit enhanced interfacial binding energies with the PEEK/PTFE matrix by 9.54% (HO-GNS), 29.87% (COOH-GNS), and 18.71% (NH_2_-GNS). These research findings indicate that the functionalized GNS can significantly enhance their interfacial interactions with the PEEK/PTFE composites, among which the COOH-GNS exhibit the best enhancement effect. Specifically, when the composite is subjected to tensile or compressive loads, a higher interfacial binding energy corresponds to a stronger resistance of GNS against detachment from the composites. This characteristic is mainly reflected in two aspects during the loading process: on one hand, the “stiffness enhancement effect” of GNS on the composite becomes more pronounced, which corresponds to a higher elastic modulus of the composite; on the other hand, stress can be uniformly transferred from the composite to GNS without obvious interfacial stress interruption, thus preventing the overall failure of the composite caused by interfacial fracture and ultimately manifesting as a higher tensile strength.

**Figure 8:**
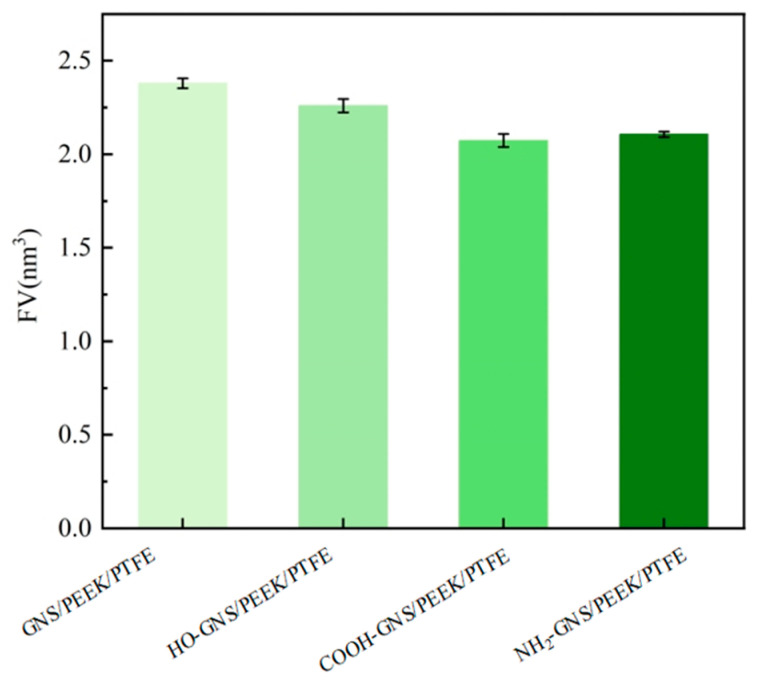
The FV of functionalized GNS/PEEK/PTFE composites.

The FV was determined to validate the hypothesis. The FV of the GNS/PEEK/PTFE composites was calculated based on the NPT molecular dynamics equilibrium trajectory file as shown in Figure 8. Specifically, the Connolly surface method was employed in this study for FV calculation, with key parameters set as follows: a probe radius of 0.1 nm and a grid spacing of 0.05 nm [43].

The FV values of HO-GNS, COOH-GNS, and NH_2_-GNS/PEEK/PTFE composites were determined to be 2.38 nm^3^, 2.26 nm^3^, and 2.07 nm^3^, respectively, as demonstrated in Figure 8. When compared to GNS (2.38 nm^3^), these functionalized composites exhibited significant reductions in FV: 5.04% (HO-GNS), 13.03% (COOH-GNS), and 11.34% (NH_2_-GNS). This indicates that the incorporation of functionalized GNS effectively compresses the free motion space of PEEK/PTFE molecular chains. The functional groups infiltrate the interior of PEEK/PTFE molecular chains, occupying residual interstitial voids and enhancing steric hindrance, restricting the conformational flexibility of PTFE and PEEK chains. H bonding interactions form between the -OH, -COOH, and -NH_2_ groups grafted on GNS and H/O/F atoms within the PEEK/PTFE matrix, significantly improving the structural stability of the composite system. COOH-GNS demonstrates the most significant reduction in FV within the PEEK/PTFE composite, aligning with the previously calculated interfacial binding energy results. This study employed atomic coordinate trajectory analysis to derive MSD values, enabling a synergistic investigation of FV dynamics and MSD behaviors to further investigate the influence of functionalized GNS on the mechanical properties of PEEK/PTFE composites.

**Figure 9:**
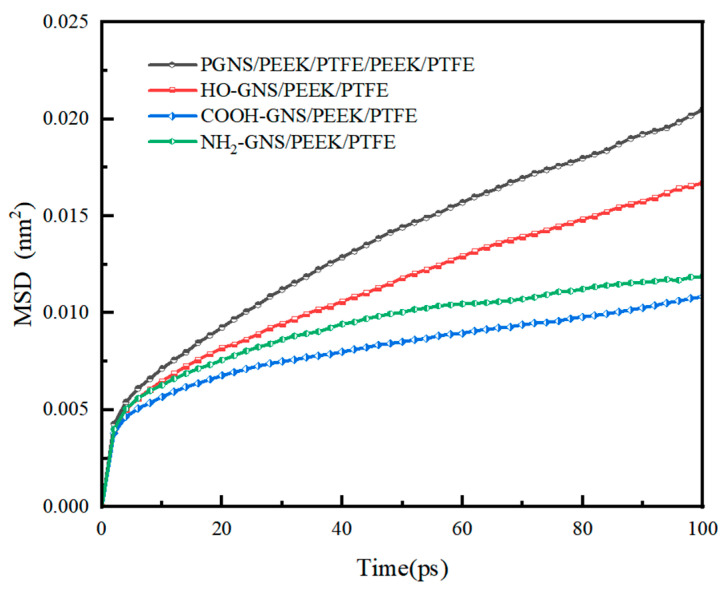
MSD curves of PEEK/PTFE chains during MD simulations.

MSD quantifies the displacement of particles over time relative to their initial reference positions, serving as a critical indicator for characterizing the collective motion dynamics of polymer chains [44]. MSD indirectly reflects the activity of molecular chains within polymer systems. A higher MSD value indicates more intense molecular chain motion, which correlates with poorer mechanical properties and thermal stability. The computational formula is presented in Equation (8).

(8) $$MSD=\frac{1}{3N}\sum_{i=0}^{N-1}\left[|R_{i}(t)-R_{i}{\left(0\right)}^{2}|\right]$$

where *R*_i_(*0*) and *R*_i_(*t*) denote the vector displacements of any atom *i* within the system at the initial time and time *t*, respectively. The MSD of the composite material system was calculated based on the NVT molecular dynamics equilibrium trajectories. The total duration of MD simulations in this study was set to 500 ps. The data corresponding to the first 100 ps was extracted for visualization in Figure 9, as the MSD curves of all systems within this time frame exhibited a stable linear growth trend, which is sufficient to effectively characterize the relative differences in molecular chain mobility among various composite systems, as illustrated in Figure 9.

The MSD values of HO-GNS, COOH-GNS, and NH_2_-GNS/PEEK/PTFE composites were determined to be 0.0116 nm^2^, 0.0083 nm^2^, and 0.0095 nm^2^, respectively, as illustrated in Figure 9. These values exhibited reductions of 16.31%, 40.11%, and 31.46% compared to PGNS (0.01386 nm^2^/ps). This indicates that the incorporation of functionalized GNS occupies more volume within the composite system, compressing the accessible space for molecular chain mobility in PEEK and PTFE, enhancing the structural stability of the composite. Furthermore, the MSD values of the composite with COOH-GNS are slightly lower compared to those with NH_2_- or HO-functionalized GNS as shown in Figure 9. This phenomenon arises from the higher polarity of -COOH anchored on the GNS surface, which establishes stronger interfacial interactions with specific atomic sites in PEEK and PTFE chains, consequently reducing segmental mobility. The MSD values of the four composite systems align with the previously discussed interfacial binding energy and FV results. Specifically, the MSD data for COOH-GNS-filled composites demonstrate significantly restricted polymer chain mobility, corroborating the enhanced interfacial interactions and reduced FV availability in these systems. We further computed the RDF for critical atomic pairs to elucidate the microscopic structural origins of these kinetic behavioral discrepancies. This analysis aims to investigate the localized interfacial interactions and spatial distribution characteristics between functionalized GNS and polymer chains.

**Figure 10:**
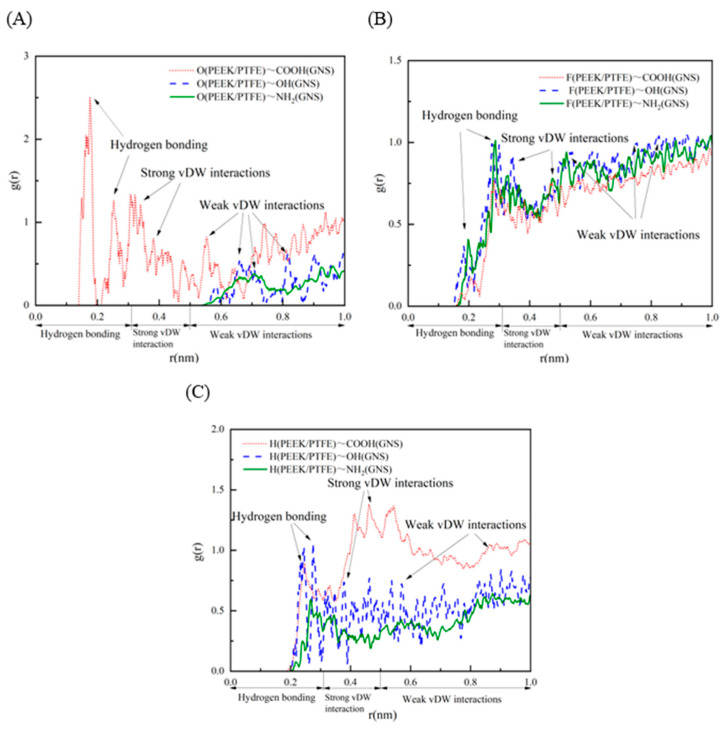
RDF values between O, F, H atoms of PEEK/PTFE chains and functional groups: (**A**) O atoms—functional groups; (**B**) F atoms—functional groups; (**C**) H atoms—functional groups.

The RDF serves as a pivotal characterization tool for analyzing local spatial correlations among atoms and microstructural distributions in polymers, which can reflect the short-range arrangement characteristics of atoms and molecular chains within composites [45,46,47,48,49,50]. To elucidate the mechanistic effects of functionalized GNS on the mechanical properties of PEEK/PTFE composites, this study computationally analyzed the atomic spatial proximity characteristics between functionalized GNS and the PEEK/PTFE matrix. Based on the NPT dynamic equilibrium trajectories, we calculated the RDFs between O, F, H atoms in PEEK/PTFE and their corresponding functional groups, with the results presented in Figure 10. By analyzing the characteristic peak positions (*r*) in the RDF curves, the spatial interaction distance ranges between atoms can be determined: characteristic peaks at *r* < 0.31 nm correspond to the typical spatial distance interval of chemical bonds or hydrogen bonds; those at 0.31 nm < *r* < 0.5 nm fall within the common distance range of strong vdW interactions; and peaks at *r* > 0.5 nm are indicative of the spatial scale of weak vdW interactions.

The RDF between O atoms and -OH groups exhibits two characteristic peaks at *r* = 0.68 nm and 0.82 nm, with peak intensities of 0.53 and 0.55, respectively, indicating that a small fraction of O atoms and -OH groups are within the spatial proximity range of weak vdW interactions. The RDF of O atoms and -NH_2_ groups shows two characteristic peaks at *r* = 0.67 nm and 0.92 nm, with peak values of 0.41 and 0.44, suggesting that the spatial distance between these two species also lies within the interval of weak vdW interactions. For the RDF of O atoms and -COOH groups, two characteristic peaks emerge at *r* = 0.19 nm and 0.32 nm, with peak intensities reaching 2.51 and 1.41, which demonstrates that the spatial distance between most O atoms and -COOH groups conforms to the typical scale of hydrogen bond interactions, while the distance between a small number of O atoms and -COOH groups corresponds to the range of strong vdW interactions as shown in Figure 10A.

The RDF between F atoms and -OH groups display two characteristic peaks at *r* = 0.29 nm and 0.33 nm, with peak values of 0.98 and 0.62, respectively, implying that the spatial distance between most F atoms and -OH groups fall within the common interval of hydrogen bond interactions. The RDF of F atoms and -NH_2_ groups has two characteristic peaks at *r* = 0.30 nm and 0.47 nm, with peak intensities of 1.01 and 0.79, indicating that the spatial proximity characteristics between them are consistent with the distance scales of hydrogen bonds and strong vdW interactions. The RDF of F atoms and -COOH groups presents two characteristic peaks at *r* = 0.29 nm and 0.46 nm, with peak values of 0.76 and 0.75, which illustrates that the distance between some F atoms and -COOH groups lies within the hydrogen bond interaction interval, and that between others corresponds to the spatial scale of strong vdW interactions as shown in Figure 10B.

The RDF results of H atoms with -OH, -NH_2_ and -COOH groups reveal that the spatial distance between some H atoms and these three functional groups conforms to the typical scale of hydrogen bond interactions, and more H atoms are within the proximity range of strong vdW interactions with -COOH groups. The -COOH group contains strongly polar O-H bonds and C=O double bonds; these polar groups tend to form short-range spatial arrangements with high-electronegativity O and F atoms, thus exhibiting distinct RDF characteristic peaks as shown in Figure 10C.

Based on the RDF analysis results of O, F, H atoms and functional groups, Table 1 systematically summarizes the atomic spatial interaction characteristics between functionalized GNS and the PEEK/PTFE matrix. It should be noted that RDF only reflects the spatial proximity of atoms, and the specific types and intensities of interactions need to be further verified by combining energy or angular analysis. The existing RDF results indicate that there are more pronounced short-range atomic arrangement characteristics between functionalized GNS and the PEEK/PTFE matrix, which provides a structural basis for the formation of interfacial interactions, thereby being conducive to improving the mechanical properties of the composites.

In summary, functionalized GNS form surface wrinkles through hybridization transition (sp^2^→sp^3^), which inhibits self-agglomeration and improves dispersion stability in the matrix from the perspective of microscopic mechanisms. Meanwhile, the functional groups grafted on the surface of GNS form stronger interfacial interactions with the matrix. These strong interfacial interactions further compress the FV of the composites (the FV of the COOH-GNS system is reduced by 13.03% compared with that of PGNS) and restrict the movement of polymer molecular chains (the MSD is decreased by 40.11% compared with that of PGNS), ultimately achieving efficient load transfer and avoiding interfacial fracture failure.

## 4. Conclusions

This study investigates the mechanical properties and underlying mechanisms of PEEK/PTFE composites reinforced with functionalized GNS. By adopting a multi-scale research framework and conducting in-depth analysis of core reinforcement mechanisms, the following innovative findings are obtained:

Experimental testing combined with MD simulations establishes an integrated research system linking macroscopic performance characterization to microscopic mechanism interpretation, overcoming the limitations of traditional experimental methods in precise analysis of interfacial interactions. Macroscopic tensile and compression tests directly verify the enhanced mechanical performance of the composites; microscopically, quantitative analysis of interfacial binding energy, FV, MSD and RDF via MD simulations elucidates the atomic-scale reinforcement mechanism of functionalized GNS and establishes a definite correlation between macroscopic properties and microscopic behaviors.

Functionalized GNS significantly improves the macroscopic mechanical properties of PEEK/PTFE composites. Compared with GNS, COOH-GNS shows the most pronounced enhancement, increasing elastic modulus, tensile strength, yield strength, and compressive modulus by 47.09%, 31.1%, 45.16%, and 20.91%, respectively. MD simulations further confirm this trend, with COOH-GNS reinforced composites exhibiting increases of 59.48% in Young’s modulus, 49% in shear modulus, and 21.57% in bulk modulus relative to the PGNS system, which aligns with experimental results.

Microscopic analysis verifies that functionalized GNS realizes mechanical reinforcement of the composites via a dual mechanism: the sp^2^→sp^3^ hybridization of surface carbon atoms induces GNS wrinkling to elevate roughness and stiffness, suppressing agglomeration and improving dispersion; functional groups strengthen interfacial interactions (with a 29.87% increase in interfacial binding energy for the COOH-GNS system), occupy matrix free volume to constrain molecular chain motion, and anchor polymer chains for efficient load transfer. The synergistic effect of these multiple factors leads to a remarkable improvement in the mechanical properties of the composites.

Through rational functional group regulation, the prepared functionalized GNS reinforced PEEK/PTFE composites achieve synergistic improvement of PTFE’s intrinsic characteristics and mechanical performance, overcoming the performance trade-off dilemma of conventional systems. This research provides a novel material strategy for harsh dynamic sealing conditions, as well as theoretical support for the rational selection of functionalized nanofillers and the modification optimization of composite materials.

Although this study has systematically explored the mechanical performance and reinforcement mechanisms of functionalized GNS-reinforced PEEK/PTFE composites, several limitations remain due to the restrictions of research conditions and technical methods. The primary limitations are as follows: the fully amorphous MD model of the PEEK/PTFE composite system ignores the semicrystalline characteristics of the two polymers, leading to the neglect of crystallinity and crystal structure effects on molecular chain arrangement and load transfer efficiency, which may reduce the accuracy of mechanical property analysis. Moreover, the relationship between the sintering process and polymer crystallinity has not been systematically clarified experimentally. Only static interfacial interactions were characterized by RDF without dynamic quantitative analyses (e.g., EDA and hydrogen bond lifetime calculations), which impedes the precise revelation of hydrogen bond formation/rupture dynamics and the role of electron cloud distribution in interfacial bonding strength. To compensate for these limitations and further understand the reinforcement mechanisms of functionalized GNS, subsequent research will carry out in-depth studies on the above-mentioned aspects.

## Figures and Tables

**Table 1:** Intermolecular interactions between functionalized GNS and PEEK/PTFE matrix.

Composite System	Primary Intermolecular Interactions	Secondary Intermolecular Interactions
HO-GNS/PEEK/PTFE	Typical distance range of weak van der Waals interactions	Typical distance interval of hydrogen bonds (minority)
COOH-GNS/PEEK/PTFE	Typical distance interval of hydrogen bonds	Common distance range of strong van der Waals interactions (minority)
NH_2_-GNS/PEEK/PTFE	Typical distance range of weak van der Waals interactions	Typical distance interval of hydrogen bonds (minority)

## Data Availability

Data will be made available on request.
